# Construction of a potential long noncoding RNA prognostic model involved competitive endogenous RNA for patients with gastric cancer

**DOI:** 10.1097/MD.0000000000038458

**Published:** 2024-06-14

**Authors:** Tianyi Xi, Yuying Zhou, Sai Ma, Wen Lu, Yibin Sun, Chunrong Sun, Yu Zhou

**Affiliations:** a Department of General Surgery, The Affiliated Suzhou Hospital of Nanjing Medical University, Suzhou Municipal Hospital, Gusu School, Nanjing Medical University, Suzhou, Jiangsu Province, China; b Department of Central Laboratory, The Affiliated Suzhou Hospital of Nanjing Medical University, Suzhou Municipal Hospital, Gusu School, Nanjing Medical University, Suzhou, Jiangsu Province, China; c Department of Gastroenterology, The Affiliated Suzhou Hospital of Nanjing Medical University, Suzhou Municipal Hospital, Gusu School, Nanjing Medical University, Suzhou, Jiangsu Province, China; d Department of General Surgery, Second Affiliated Hospital of Soochow University, Souzhou, Jiangsu Province, China.

**Keywords:** bioinformatics, competing endogenous RNA network, gastric cancer, long noncoding RNA, prognosis risk model, the cancer genome atlas

## Abstract

Increasing evidence has underscored the role of long noncoding RNAs (lncRNAs) make up the major proportion of the competing endogenous RNAs (ceRNAs) network and can regulate gene expression by competitively binding to miRNAs in the development and progression of tumors. Nevertheless, the role of lncRNA-mediated ceRNAs in gastric cancer (GC) and their regulatory mechanisms have not been elucidated to some extent. This study is aimed at constructing a prognostic risk model for GC based on lncRNAs. A TCGA (The Cancer Genome Atlas) dataset was analyzed using edgeR to identify differentially expressed lncRNAs (DElncRNAs) in GC tissues vs normal tissues. Subsequently, DElncRNAs that could predict GC prognosis were determined using a training set. A prognostic risk model based on the DElncRNAs was then constructed. The performance of the model was tested using a test set. The functions of these lncRNAs in GC were investigated using a lncRNA-miRNA-mRNA network. Analysis of lncRNA expression in 407 TCGA GC cases identified 3 lncRNAs that significantly correlated with prognosis. GC cases with high-risk scores showed markedly poor prognosis relative to those with low-risk scores in both the training and test sets. Univariate and multivariate Cox regression analysis of the relationship between various clinical features and prognosis found that these lncRNAs and stage significantly correlated with GC prognosis. A lncRNA-miRNA-mRNA network based on 3 lncRNAs and functional enrichment analysis of interacting mRNA indicated that these genes are enriched in various intracellular receptor signaling pathways, including regulation of muscle system process, and protein deubiquitylation. The current study provides novel insights into the lncRNA-related ceRNA network in GC and sheds lights on underlying 3 lncRNA biomarkers may be independent prognostic signatures in predicting the survival of GC patients.

## 1. Introduction

In China, gastric cancer (GC) is one of the most prevalent cancers.^[1,2]^ GC is often diagnosed late and by the time of diagnosis, more than 60% of the patients have regional or distant metastases.^[2]^ The 5-year GC survival largely depends on tumor stage at the time of diagnosis, treatment, and several prognostic factors. Tumor-node-metastasis, the main system for predicting patient prognosis, is characterized by limited clinical prediction information.^[3]^ Thus, more reliable indicators of GC prognosis and therapeutic targets are urgently needed.

LncRNA, noncoding RNA with a length of about 200 nucleotides, are regarded as potential markers for early screening and prognosis prediction.^[4–6]^ Numerous studies have shown that lncRNAs modulate tumor biology in a variety of pathways, thereby affecting gene expression.^[5,7]^ A recent study found that lncRNA MNX1-AS1 upregulation in GC correlates with poor prognosis.^[8]^ lncRNAs are also implicated in chemotherapy resistance.^[9,10]^ The concept of competitive endogenous RNA (ceRNA) was advanced by Salmena et al,^[11]^ depicting a unique mode of interplay involving noncoding RNA (ncRNA) and mRNA within cellular regulation. Long noncoding RNAs (lncRNAs), possessing miRNA response elements (MREs) functioning as competing endogenous RNAs (ceRNAs), are significant regulators integral to diverse pathological conditions such as tumor genesis. miRNAs, demonstrated to exhibit abnormal expression in numerous malignancies, exert influence on posttranscriptional transcripts.^[12]^ Various studies have assessed correlation between lncRNA expression and GC progression and metastasis.^[13,14]^ Nevertheless, our comprehension of the correlation between lncRNA-miRNA-mRNA ceRNA networks and their constituents in delineating the prognosis of GC remains exceedingly rudimentary.

Thus, accurate GC diagnostic, prognostic and therapeutic tools are urgently needed. Here, we analyzed a lncRNA expression dataset from the Cancer Genome Atlas (TCGA) and identified differentially expressed lncRNAs (DElncRNAs) in GC versus control tissues using univariate and multivariate Cox analysis and receiver operating characteristic curve (ROC) survival analysis. This analysis identified 3 lncRNAs that significantly correlated with GC prognosis. Utilizing this innovative methodology for forecasting cancer-associated lncRNA and ceRNA networks can illuminate the intricate lncRNA-assisted ceRNA regulatory processes in GC progression and clinical course, paving way to newly discernible lncRNAs as promising diagnostic indicators or therapeutic focal points.

## 2. Materials and methods

### 2.1. Collection and grouping of GC data

Gene expression datasets and associated clinical records from GC patients were downloaded from TCGA (https://portal.gdc.cancer.gov) on March 1, 2021. The dataset contained gene expression profiles of 375 GC tissues and 32 normal tissues. Clinical information of cancer samples is shown on Supplementary Table 1a, http://links.lww.com/MD/M816.

### 2.2. Differential expression analysis

First, expression data on 12299 lncRNAs was retrieved from the RNAseq data. EdgeR limma package was then used to identify DElncRNAs between 373 GC tissue samples and 32 normal tissue controls using *P ≤* .05 and |log2FC| > 1 as cutoff thresholds. Data was visualized on volcano edgeR “ggplot2” package.

### 2.3. DElncRNAs target genes and target genes functional enrichment analysis

mRNA that interact with various lncRNAs were identified using the lncRNA-mRNA interaction relationship on RAID2.0 (http://www.rna-society.org/raid2.0/index.html). EdgeR “ClusterProfiler” package was used to determine pathway enrichment and function. Significantly enriched pathways were indicated by *P ≤* .05 as cutoff threshold.

### 2.4. Building risk model

The cancer cases were randomly divided into a training and test set at a 1:1 ratio. Univariate Cox regression analysis of the training set identified genes that significantly correlated with prognosis (*P ≤* .05) and the coefficient of each lncRNA determined by multivariate Cox regression analysis and used to construct the prognosis model. Risk score, the sum of each key lncRNA multiplied by the corresponding weight, is given by the formula:


$$\begin{array}{l} Riskscore=\sum EXP*coef. \end{array}$$

### 2.5. Efficacy analysis and validation of prognosis model

The surv_cutpoint function was applied on the training and the test set to determine the optimal threshold point for patients with low- and high-risk GC. Kaplan–Meier survival analysis was used to evaluate the prognostic model ability to predict OS. EdgeR Survival ROC package was used to predict 1-, 3-, and 5-year survival.

### 2.6. Prognostic efficacy of clinicopathological features

Univariate and multivariate Cox regression analysis was used to evaluate the prognostic value of various clinical and pathological features, including Riskscore, tumor stage, and tumor grade in the training and test sets.

### 2.7. Nomogram construction

Nomograms are based on multivariate Cox regression analysis. They integrate multiple prediction indexes and draw a line segment with scale on the same plane according to a certain proportion to express relationships between variables in the prediction model. To predict 1-, 3-, and 5-year survival, we constructed a nomogram using edgeR “Rms” package was.

### 2.8. Construction and functional enrichment analysis of a IncRNA-miRNA-mRNA network

For the 3 core lncRNAs included in the above risk model, LncBOOk (https://bigd.big.ac.cn/lncbook) was used to identify interacting miRNAs. Starbase (https://www.starbasegame.com/) was then used to identify lncRNAs target mRNAs. Based on the interaction relationship of 3 core lncRNAs, a lncRNA-miRNA-mRNA network was constructed and visualized by Cytoscape. The function of the mRNAs in the network were evaluated using edgeR “clusterProfiler” package, and enrichment pathways analyzed (*P ≤* .05).

## 3. Results

### 3.1. Differentially expressed lncRNAs

**Figure 1.**
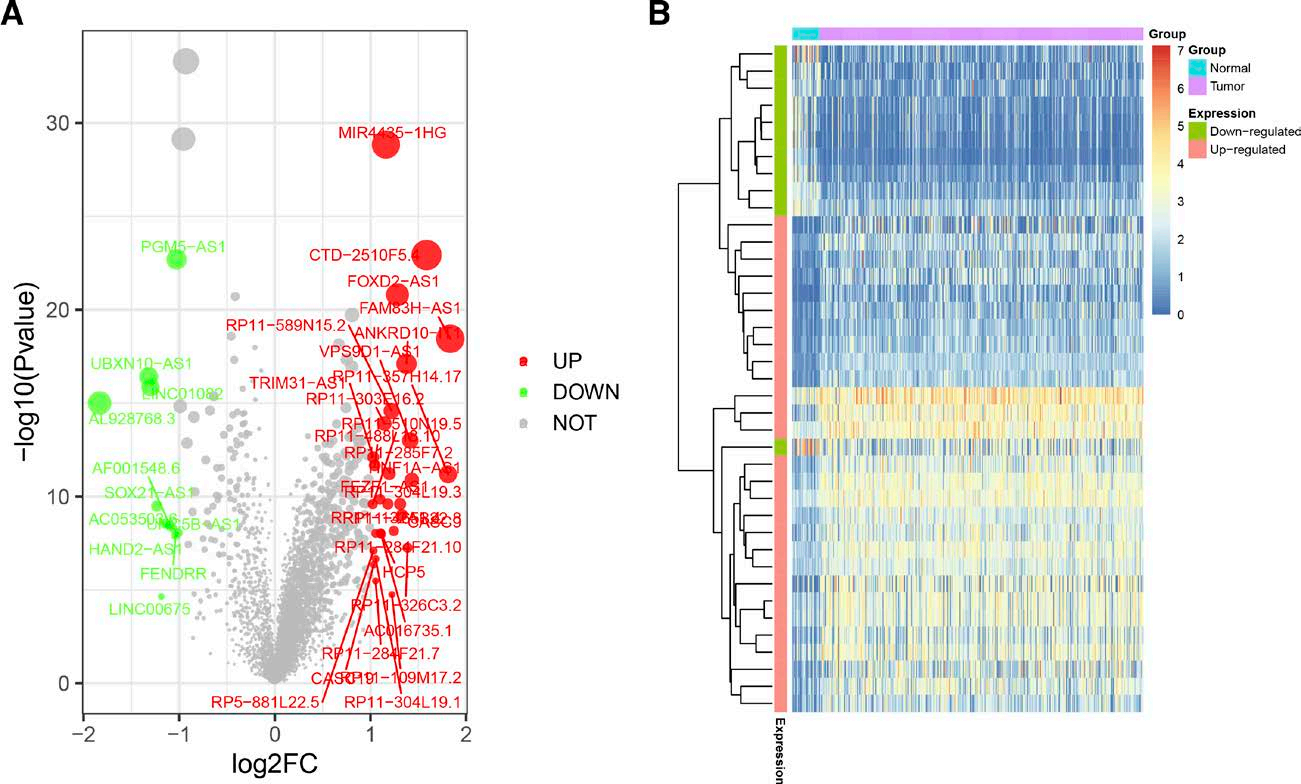
The differentially expressed lncRNA of gastric cancer. (A) Volcano map. (B) Differential gene heat map, row is sample and column is gene (|log2FC| >1 and *P* < .05). lncRNAs = long noncoding RNAs.

EdgeR limma package was used to identify differentially expressed genes in 373 GC samples vs 32 normal controls. According to the threshold, 39 DElncRNAs were obtained of these, 11 lncRNAs were poorly expressed in cancer tissues and 28 were highly expressed (Fig. 1, Supplementary Table 1b, http://links.lww.com/MD/M816).

### 3.2. DElncRNAs target genes and target genes functional enrichment analysis

**Figure 2.**
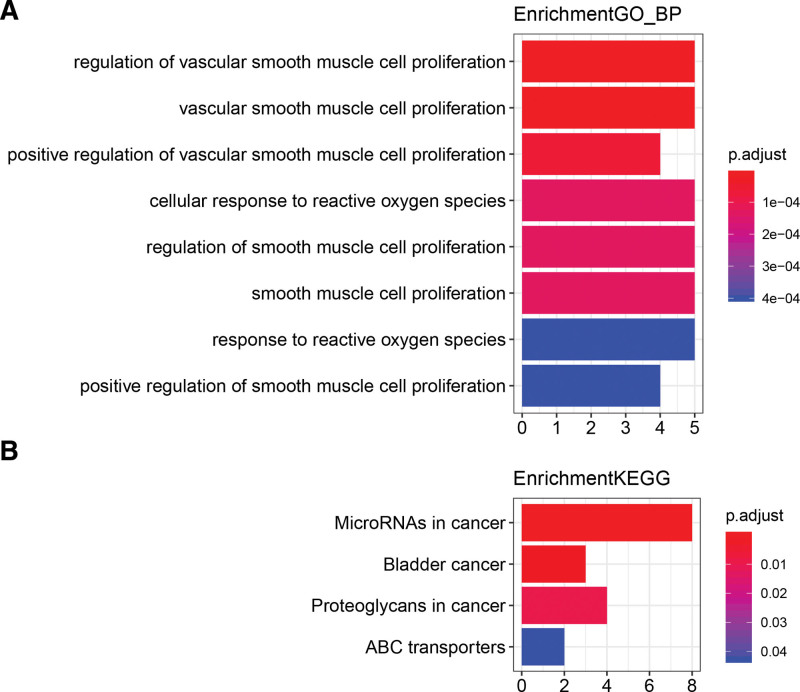
The results of DElncRNAs pathway enrichment analysis. (A) The results of gene ontology analysis. (B) The results of KEGG pathway analysis. *P* < .05 is statistically significant. KEGG = Kyoto Encylopedia of Genes and Genomes.

The lncRNA-mRNA interaction relationship on RAID2.0 database (http://www.rna-society.org/raid2.0/index.html) identified 20 target mRNAs that interacted with various lncRNAs (Supplementary Table 2, http://links.lww.com/MD/M817). Next, EdgeR “clusterProfiler” package was used for pathway enrichment analysis with *P ≤* .05 indicating significantly enriched functions and pathways. Finally, there was no significant enrichment in GOCC pathway. There was only 1 significant enrichment in metabolic pathway. However, there were 4 significant enrichments in Kyoto Encylopaedia of Genes and Genomes (KEGG) pathway. Therefore, only GO_BP and KEGG pathways are shown. This analysis revealed that the target genes were significantly enriched in cancer related pathways, including MicroRNAs in cancer, bladder cancer and proteoglycans in cancer (Fig. 2, Supplementary Table 3, http://links.lww.com/MD/M818).

### 3.3. Building risk model

**Figure 3.**
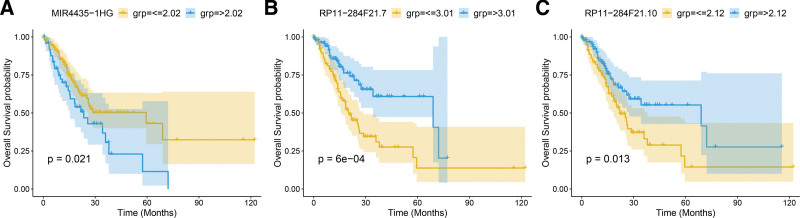
The overall survival curves of 3 prognosis-related lncRNA. (A–C) lncRNA (MIR4435-1HG), RP11 − 284F21.7, RP11 − 284F21.10. *P* < .05 is statistically significant. lncRNAs = long noncoding RNAs.

**Figure 4.**
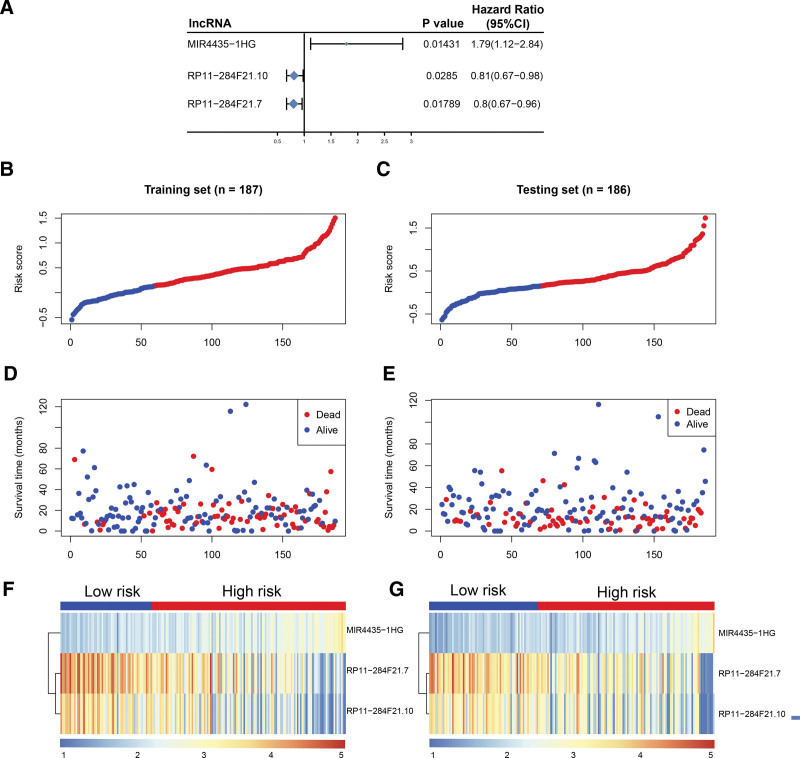
The risk score of 3 prognosis-related lncRNA. (A) Forest map of 3 prognosis-related lncRNA. (BcC) Risk score distribution of training set and test set. (D–E) The relationship between survival time and sample state in training set and test set. (F–G) Expression of 3 prognosis-related lncRNA in training set and test set. *P* < .05 is statistically significant. lncRNAs = long noncoding RNAs.

First, all cancer samples were randomly divided into a training (n = 187) and test set (n = 186) at a 1:1 ratio. The training set was then used to build a risk model (Supplementary Table 4, http://links.lww.com/MD/M819) and univariate Cox regression analysis used to identify lncRNAs that significantly correlated with prognosis (*P ≤* .05, Supplementary Table 5, http://links.lww.com/MD/M820). Using surv_cutpoint function to find the best threshold, the 3 prognosis-related lncRNAs were subjected to Kaplan–Meier analysis (Fig. 3). Multivariate Cox regression analysis was then used to assess the effect of various lncRNA on prognosis, i.e., lncRNA $coef\;f\;icient^{coef}$. Finally, risk score was given by the sum of each key lncRNA multiplied by the corresponding weight (Riskscore = Exp(MIR4435-1HG)*0.561 + Exp(RP11-284F21.7)*(-0.338) + EXP(RP11-284F21.10)*0.136). Based on the risk model, risk scores were calculated for training and test set samples (Supplementary Table 6, http://links.lww.com/MD/M821). Using the surv_cutpoint, samples were divided into a high and low-risk group (Fig. 4), the risk score of training set and test set.

### 3.4. Effectiveness analysis and validation of prognosis model

**Figure 5.**
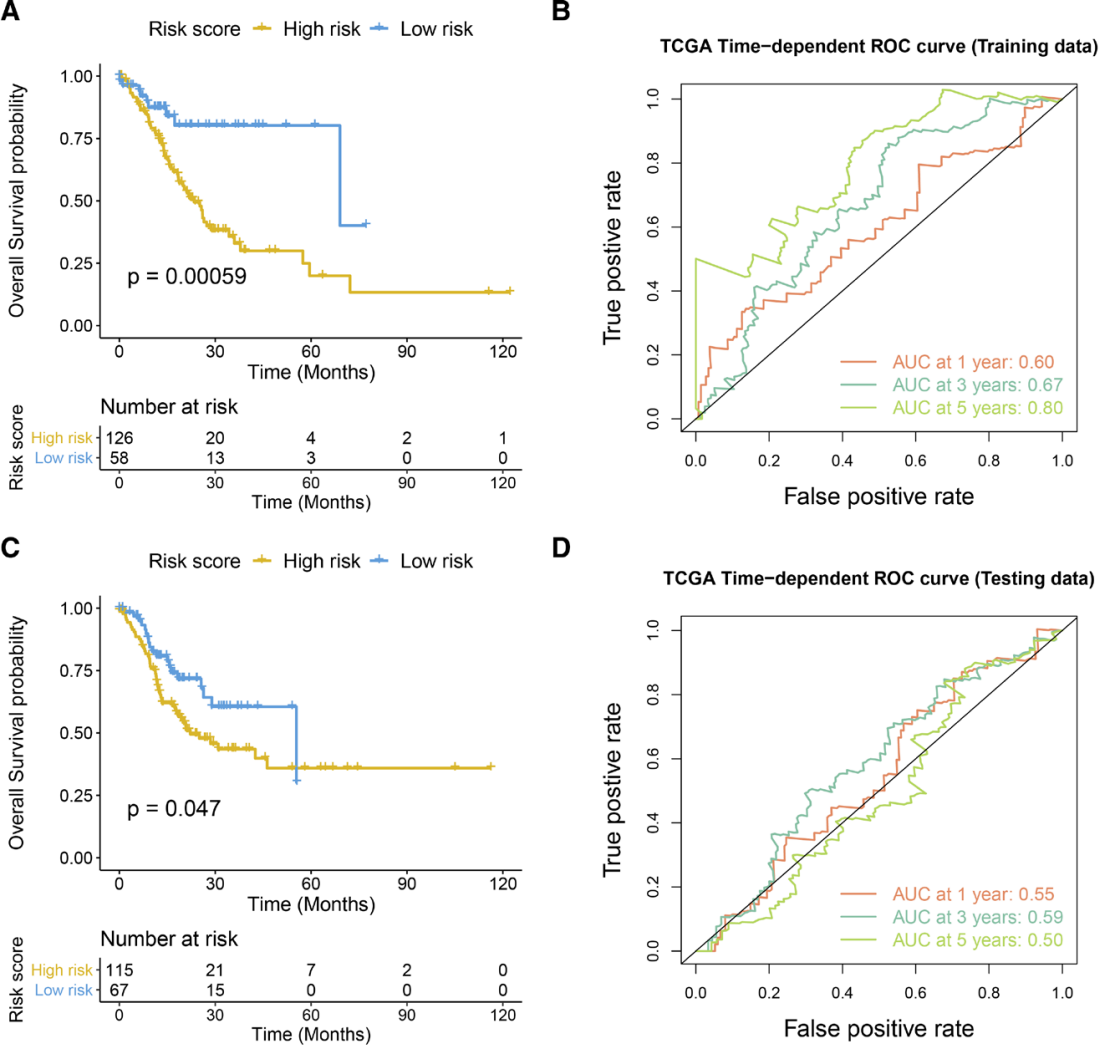
The effectiveness analysis and verification of prognostic model. (A–B) Training set: K-M survival curve (OS) and ROC curve. (C–D) Test set: K-M survival curve (OS) and ROC curve. *P* < .05 is statistically significant. OS = overall survival, ROC = receiver operating characteristic curve.

Based on training and test set risk scores and the corresponding high and low-risk group information, the predictive ability of the prognosis model was evaluated using Kaplan–Meier survival analysis. This analysis showed that OS and disease free survival of the low-risk group was significantly better than that of the high-risk group in the training set. ROC curve analysis of 1-, 3-, and 5-year survival showed that the risk model had good prognostic effect. Similarly, in the test set, OS of the low-risk group was significantly better than in the high-risk group, and the ROC curve indicated that the risk model had good prognostic effect (Fig. 5).

### 3.5. Prognostic efficacy of clinicopathological features

**Figure 6.**
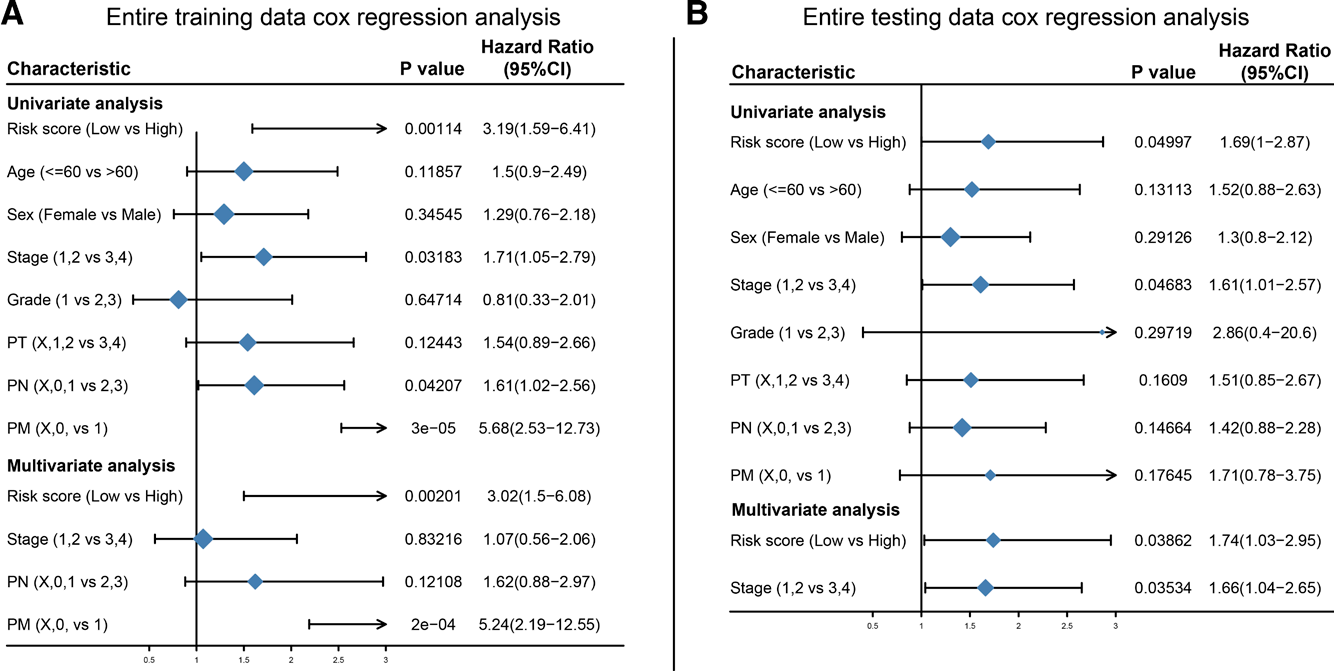
The prognostic efficacy analysis of characteristics. (A) Univariate and multivariate Cox regression analysis of clinical characteristics in training set. (B) Univariate and multivariate Cox regression analysis of clinical characteristics in test set. *P* < .05 is statistically significant.

For the clinical features of training set and test set, such as Riskscore, tumor stage, tumor grade, etc. Univariate Cox regression analysis revealed that Riskcore, stage, pM, and pN significantly correlated with prognosis in training set. In the test set, Riskscore and stage significantly correlated with prognosis. Multivariate Cox regression analysis of Riskcore, stage, pM, and pN on the training set revealed that Riskscore and pM still significantly correlated with prognosis. Similarly, multivariate Cox regression analysis on the test set revealed that Riskscore and stage significantly correlated with prognosis (Fig. 6).

### 3.6. Nomogram construction

**Figure 7.**
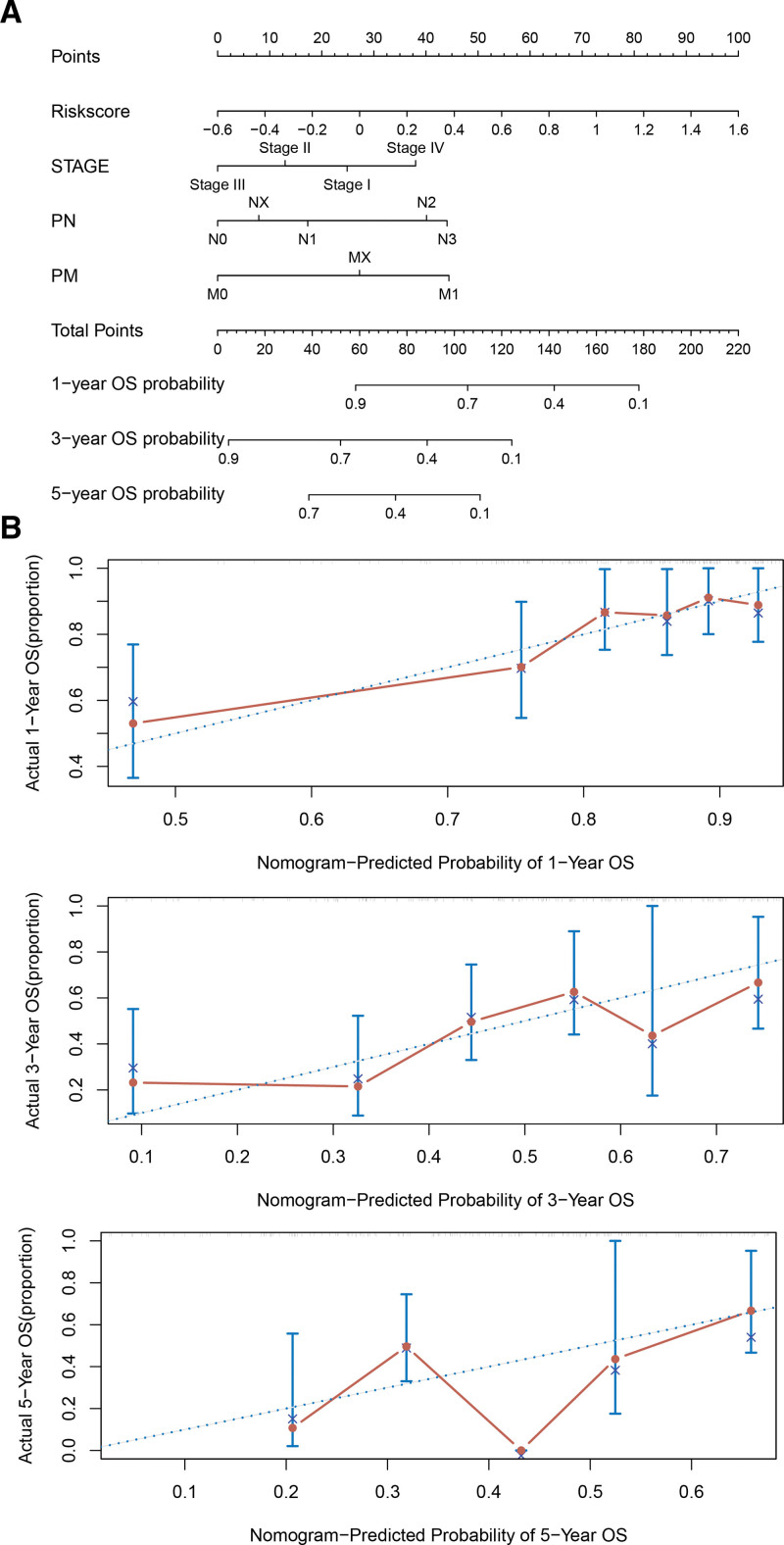
The columnar plots of clinical characteristics significantly associated with risk score and prognosis. (A) The points of stage, pM, and pN and the risk score of 1-, 3-, and 5-yr OS. (B) The result of nomogram − predicted probability of 1-, 3-, and 5-yr OS. OS = overall survival.

To predict 1-, 3-, and 5-year survival, we constructed a nomogram based on multivariate analysis. To provide clinicians with a quantitative way of predicting GC prognosis, we used the training set to construct a nomogram of clinical features like stage, pM, and pN, which correlated with prognosis (Fig. 7).

### 3.7. Construction and functional enrichment analysis of IncRNA-miRNA-mRNA network

**Figure 8.**
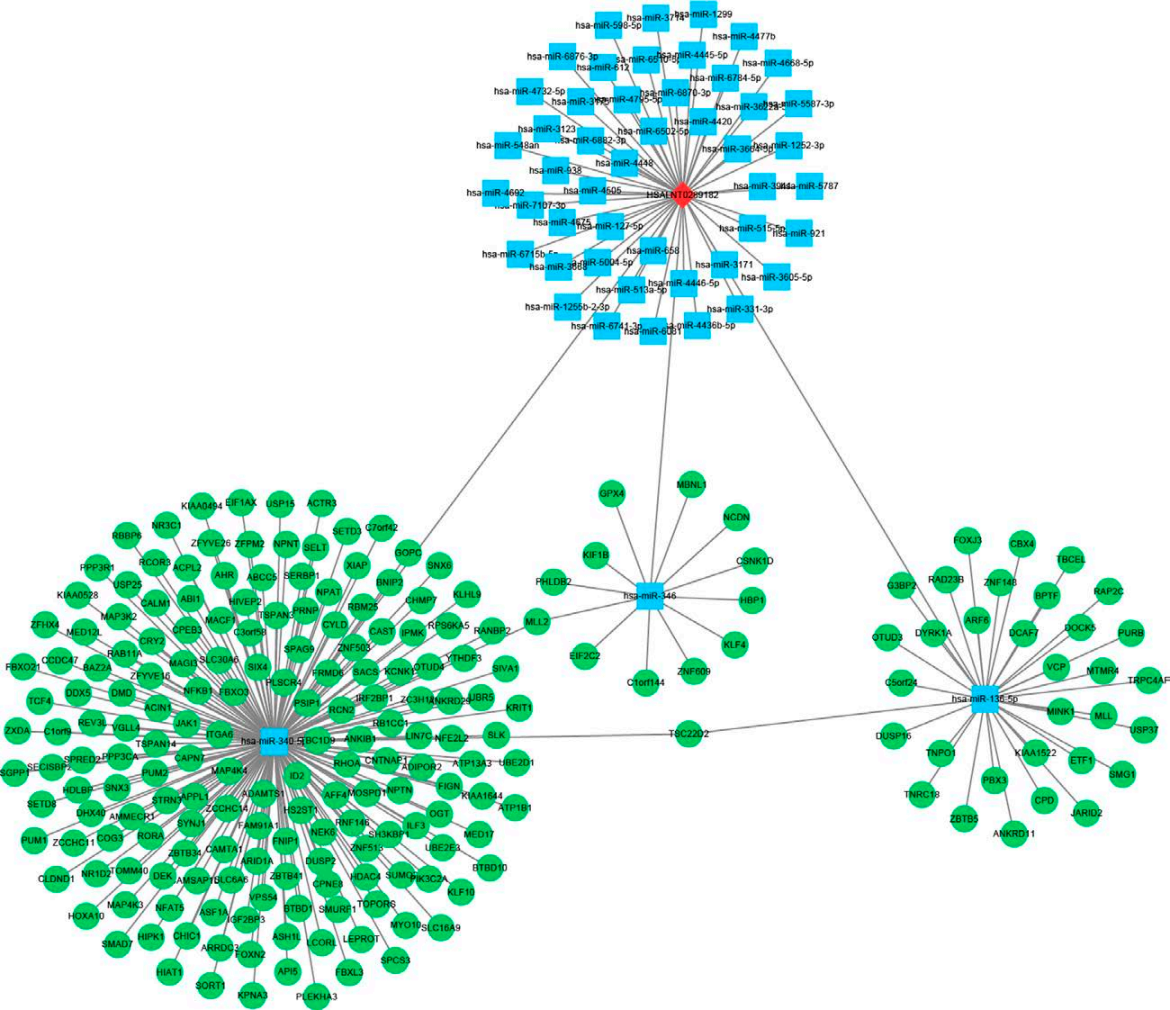
The interaction network of lncRNA-miRNA-mRNA. The red node is lncRNA (MIR4435-1HG), the blue node is miRNA and the green node is mRNA. lncRNAs = long noncoding RNAs.

To determine the potential role of these 3 lncRNAs in GC, we constructed a lncRNA-miRNA-mRNA network. For the 3 core lncRNAs included in the above risk model, LncBOOK was used to identify interacting miRNAs. Here, only MIR4435-1HG was an interacting miRNA. The top 50 miRNAs were selected based on the score of the pair (Supplementary Table 7-lncRNA-miRNA, http://links.lww.com/MD/M822). Next, Starbase revealed that these 50 miRNAs targeted 220 mRNAs (Supplementary Table 7-miRNA-mRNA, http://links.lww.com/MD/M822). Finally, a ceRNA network comprised of 1 lncRNA, 50 miRNAs, and 220 mRNA was obtained (Fig. 8, Supplementary Table 7-ceRNA, http://links.lww.com/MD/M822).

**Figure 9.**
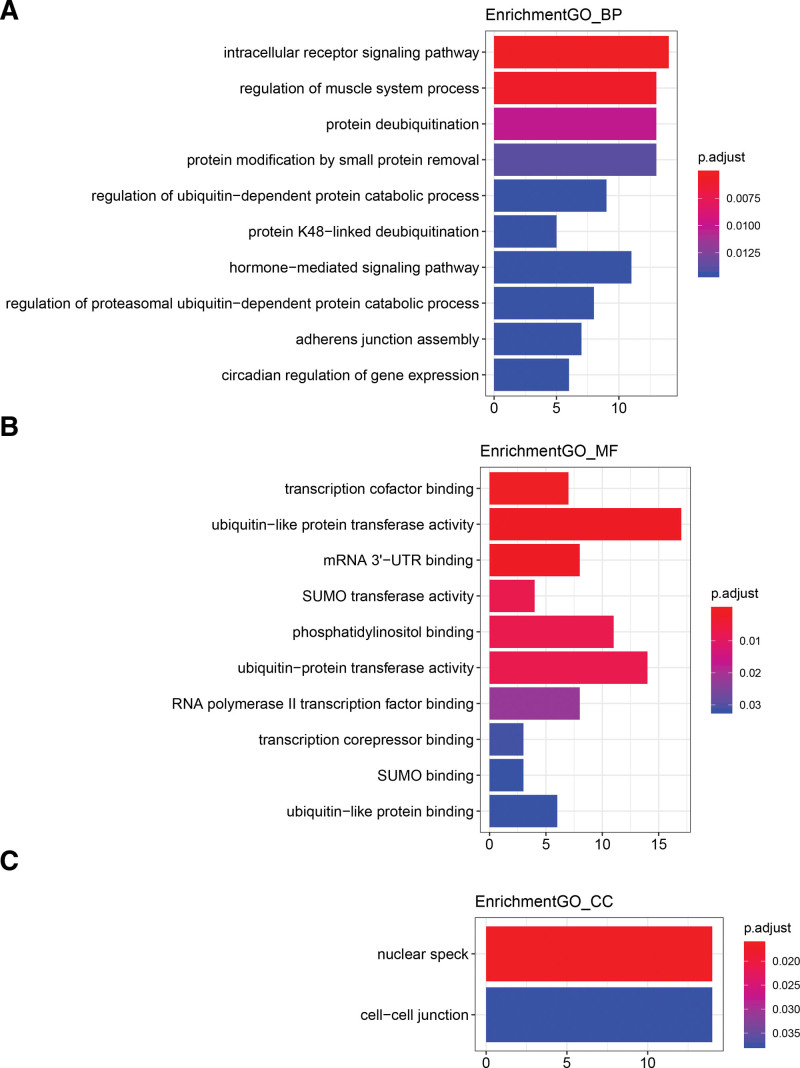
The results of mRNA in Interaction network pathway enrichment analysis. (A–C) The results of gene ontology analysis. *P* < .05 is statistically significant.

To determine their role GC progression, edgeR “clusterProfiler” package was used to analyze which pathways the 220 mRNA in the network were enriched for. With *P ≤* .05 as cutoff threshold, the number of GO-BP, GO-MF and GO-CC pathways was 58, 24, and 2, respectively. However, there was no significant enrichment of KEGG pathway (Supplementary Table 8, http://links.lww.com/MD/M823). These mRNAs were mainly enriched for intracellular receptor signaling pathway, regulation of muscle system process, and protein deubiquitylation (Fig. 9).

## 4. Discussion

GC treatment is limited and the past few decades have brought little progress in traditional and novel treatments. GC is still leading cause of morbidity and mortality worldwide. To reduce the socioeconomic burden related to GC, it is very important to identify and manage those at high-risk for GC. Thus, effective biomarkers are urgently needed to improve GC diagnosis and prognosis. Some known tumor biomarkers, like carcinoembryonic antigen, CA50, and CA724, have limited use in GC diagnosis.^[15,16]^

With the development of high-throughput sequencing technology, people can conduct in-depth research on noncoding genome on a large scale, and more and more functions of lncRNAs are gradually revealed. LncARNAs are mainly involved in many important biological functions such as cell proliferation, survival, apoptosis and movement by regulating gene expression in epigenetic, transcriptional and post transcriptional.^[17]^ At present, more and more evidence shows that the abnormalities of lncRNAs, such as overexpression, deletion or mutation, play a driving role in the malignant biological behavior of tumors, such as tumor formation, progression, metastasis and recurrence.^[18,19]^ For example, lncRNA HOTTIP upregulation in MDR GC cells has been shown to promote EMT by suppressing ZO1 and E-cad expression and elevating the expression of Twist, ZEB1 and N-cad.^[20,21]^ The lncRNA BCAR4 (breast cancer anti-estrogen resistance 4) was earlier demonstrated to the abundant in DDP-resistant GC cells. Evidence shows that BCAR4 increases expression of genes that drive cell stemness (KLF4, SOX2, and OCT3/4) by activating the Wnt/β-catenin pathway, thereby enhancing DDP resistance and stemness of GC cells.^[22,23]^ Wang et al demonstrated that expression of the lncRNA ROR (a reprogramming regulator) positively correlates with poor prognosis and MDR in GC patients.^[24]^ These reports highlight the feasibility of lncRNAs to diagnose and predict the prognosis of GC. Because lncRNAs in circulation or in GC tissue are non-invasively obtainable, they are ideal markers for early diagnosis, screening, and prognosis prediction of GC. Moreover, serum collection at various time points can conveniently monitor GC progression.^[25,26]^ Notably, the amount of lncRNAs in serum matches the level of such lncRNAs in primary tumor tissues, hence can also reflect tumor features.^[27,28]^ Here, using TCGA lncRNA expression data and clinicopathological features from GC patients, we identified and validated 3 prognosis-related lncRNAs.

A recent study found that lncRNA MNX1-AS1 (MNX1 antisense RNA 1) expression is markedly upregulated in GC tissues.^[8,29]^ Here, we identified 39 DElncRNAs in GC tissues vs normal tissues. Furthermore, 3 DElncRNAs (MIR4435-1HG, RP11-284F21.7 and RP11-284F21.10) that significantly correlated with GC prognosis were identified. Multivariate Cox regression analysis was used to construct a risk model based on the 3 lncRNAs. Xu et al found that elevated serum levels of lncRNA MIAT in GC patients correlate with poor prognosis.^[30]^ To determine the clinical feasibility of the lncRNAs, we compared them to clinical indexes of GC patients, including pathological stage, pM and pN using ROC curve survival analysis and 1-, 3-, and 5-year prognosis analyses in the training and test sets. These analyses indicated that the 3 lncRNAs may have independent prognostic value in GC and that the risk model had excellent prognostic efficacy.

It has become increasingly clear that several RNAs, lncRNAs, and ncRNAs can act as ceRNAs, to regulate miRNA expression via miRNA response elements (MREs).^[11,31]^ LncRNA-miRNA-mRNA networks have been linked to the development of GC. In GC, numerous ceRNAs that function as sponges have been reported. For example, KCNQ1OT1, a miR-504 sponge that targets cyclin-dependent kinase 16 in liver cancer and cyclin E2, a miR-370 sponge in glioma act as ceRNA by neutralizing miR-9. This promotes GC progression by regulating Lim homeobox transcription factor 1 expression.^[32,33]^ Here, a lncRNA-miRNA-mRNA interaction network based on the above 3 RNAs indicated that they are involved in transcription factor binding, suggesting that they indirectly affect gene expression.

In conclusion, based on a large dataset, we constructed a GC prognosis model based on 3 lncRNAs. Test and training set analyses show that the model can better predict GC prognosis. However, no public data in other platforms was available for independent validation of the risk model.

## Author contributions

**Data curation:** Wen Lu.

**Formal analysis:** Yibin Sun, Yu Zhou.

**Funding acquisition:** Yu Zhou.

**Methodology:** Sai Ma.

**Resources:** Yu Zhou.

**Software:** Chunrong Sun.

**Writing – original draft:** Tianyi Xi.

**Writing – review & editing:** Yuying Zhou.

## Supplementary Material
















